# Exploring Determinants of HIV/AIDS Self-Testing Uptake in South Africa Using Generalised Linear Poisson and Geographically Weighted Poisson Regression

**DOI:** 10.3390/healthcare11060881

**Published:** 2023-03-17

**Authors:** Emmanuel Fundisi, Simangele Dlamini, Tholang Mokhele, Gina Weir-Smith, Enathi Motolwana

**Affiliations:** 1 Geospatial Analytics Unit, eResearch Knowledge Centre, Human Sciences Research Council, Pretoria 0002, South Africa; 2 Geography, Archaeology and Environmental Studies, Wits University, Johannesburg 2050, South Africa

**Keywords:** HIV/AIDS, self-testing uptake, Generalised Linear Poisson Regression, Geographic Weighted Poisson Regression, SABSSM V, South Africa

## Abstract

Increased HIV/AIDS testing is of paramount importance in controlling the HIV/AIDS pandemic and subsequently saving lives. Despite progress in HIV/AIDS testing programmes, most people are still reluctant to test and thus are still unaware of their status. Understanding the factors associated with uptake levels of HIV/AIDS self-testing requires knowledge of people’s perceptions and attitudes, thus informing evidence-based decision making. Using the South African National HIV Prevalence, HIV Incidence, Behaviour and Communication Survey of 2017 (SABSSM V), this study assessed the efficacy of Generalised Linear Poisson Regression (GLPR) and Geographically Weighted Poisson Regression (GWPR) in modelling the spatial dependence and non-stationary relationships of HIV/AIDS self-testing uptake and covariates. The models were calibrated at the district level across South Africa. Results showed a slightly better performance of GWPR (pseudo R^2^ = 0.91 and AICc = 390) compared to GLPR (pseudo R^2^ = 0.88 and AICc = 2552). Estimates of local intercepts derived from GWPR exhibited differences in HIV/AIDS self-testing uptake. Overall, the output of this study displays interesting findings on the levels of spatial heterogeneity of factors associated with HIV/AIDS self-testing uptake across South Africa, which calls for district-specific policies to increase awareness of the need for HIV/AIDS self-testing.

## 1. Introduction

Sub-Saharan Africa accounts for 70% of Human Immunodeficiency Virus/Acquired Immunity Deficiency Syndrome (HIV/AIDS) infections around the globe [1]. This is despite coordinated efforts from different stakeholders that continue to scale up prevention and treatment programmes. Such programmes have resulted in an increased number of people receiving antiretroviral drugs [2,3,4]. However, poor quality of services, limited access to services, and inadequate resources tailor-made to combat the epidemic continue to undermine responses to HIV/AIDS [4,5]. According to the fifth South African National HIV Prevalence, HIV Incidence, Behaviour and Communication Survey of 2017 (SABSSM V), approximately 7.9 million people are living with HIV/AIDS in South Africa [6]. One disturbing observation noted from the national level community survey is that HIV/AIDS exceedingly affects women compared to men and it is unequally distributed across different levels of socioeconomic status [3]. To a greater extent, the socioeconomic status of women and unequal gender power structures coerce and lead women into unintended relationships that expose them to elevated risks of HIV/AIDS infection in comparison to men [5]. Similar findings have been observed in the East, West, and Central African regions where most women are mostly affected by HIV/AIDS [1].

Findings from the 2017 SABSSM survey also highlighted a higher prevalence of HIV/AIDS, and this was noted to have been perpetuated by social inequalities in South Africa, with most blacks living in poverty [3]. Similar observations and remarks were also reported by Pengpid et al. and Mabaso et al. [7,8] who noted poverty as an overarching factor that increases the disparity of HIV/AIDS prevalence among racial groups in South Africa. Correspondingly, Nehl et al. [9] observed higher HIV/AIDS prevalence amongst African–American communities in the United States of America and associated such findings with socioeconomic inequities, high levels of poverty, social exclusion, and above all, limited access to healthcare. As research and various initiatives continue to expand, finding new innovative approaches to help alleviate ills brought about by the HIV/AIDS epidemic, and delivering effective prevention programs across spatial divides, continue to be a challenge.

Extant literature investigated the variation in HIV/AIDS prevalence based on human perception, behaviour patterns, attitudes, norms, and stereotypes e.g., [4,5,7,10,11,12,13]. Focusing on people’s disability status using 2012 SABSSM data, Pengpid et al. [7] investigated HIV/AIDS prevalence, human behaviours, knowledge, and attitudes. The study utilised multivariable logistic regression and reported high HIV/AIDS stigmatisation of people with disability (Odds Ratio (OR): 0.31, Confidence Interval (CI): 0.25, 0.67) compared to people without disabilities (OR: 0.57, CI: 0.34, 0.96). Furthermore, a higher prevalence of HIV/AIDS infections was recorded from individuals with hearing impairment. Zungu et al. [6] associated people’s sexual partners, condom use during the last sexual activity, consistency of condom use and antiretroviral therapy (ART) exposure. Using logistic regression (OR = 0.6 (95% CI: 0.4–0.8), *p* = 0.001), the study reported that HIV/AIDS positive respondents on ART were less likely to have numerous sexual partners, thus adopting condom use in sexual encounters compared to HIV/AIDS respondents who were not on ART. HIV/AIDS-infected adults on ART often shifted their sexual behaviours in response to their HIV/AIDS-positive status. Analysing relationships between HIV/AIDS and socioeconomic issues between 2011 and 2016 in Sub-Saharan African countries, Sun et al. [1] utilised Poisson regression, and spatiotemporal autoregressive models. Results from the study showed significant spatiotemporal non-stationarity and autocorrelation between HIV/AIDS and socioeconomic factors. A well rounded understanding of human behaviours, and attitudes as well as spatial patterns of HIV/AIDS prevalence across the spatial divide can influence positive public health decisions by the responsible authorities, thus enhancing prioritisation of worst-affected areas [12].

Allocation of resources for HIV/AIDS testing to areas of greatest need is key to controlling the epidemic. Most importantly, it is worth noting that people with HIV infection are generally asymptomatic for several years before the virus progresses to AIDS [14]. The introduction of the 95-95-95 targets initiative by the United Nations, where 95% of HIV-infected people are meant to be cognisant of their HIV/AIDS status by 2030, is an essential global programme. In South Africa, the launch of nationwide HIV/AIDS testing initiatives, including HIV testing and counselling (HTC) in 2010 and the HTC revitalisation strategy in 2013 [11] resulted in the increased uptake of much needed antiviral drugs. Despite such programmes and initiatives (HIV/AIDS testing), millions of people continue to get affected by the HIV/AIDS epidemic and most importantly, are still oblivious to their HIV status.

Various studies have explored the association of HIV testing uptake with spatial patterns, socioeconomic status, and demographics, etc., [2,3,4,7,11,15,16,17,18,19,20,21,22,23]. For instance, Ntsepe et al. [4] analysed people’s perceptions of HIV testing in formal and informal urban communities from different races and age groups in select towns (Cape Town and Durban) across two South African provinces. Findings from the study highlighted that most respondents were afraid of testing HIV positive, and thus have misconceptions about the risks associated with HIV testing. In addition, most respondents were afraid of stigmatisation, with the fear of discrimination by society if found HIV positive. Harichund et al. [17] assessed the influence and acceptability of HIV self-testing in KwaZulu-Natal, South Africa. Using a purposive sampling survey, the study results revealed higher HIV/AIDS testing uptake among women compared to a considerably low uptake amongst men who only tested for HIV/AIDS due to convenience. Jooste et al. [11] utilised a global, Generalised Linear model to assess variations in HIV/AIDS testing uptake in South Africa, reporting variation across the country. There is a need to examine the strength and weaknesses of covariates in modelling HIV/AIDS testing uptake in different districts since one variable may have a strong influence in one district but exhibit a weaker influence in a different district.

It is therefore important to find factors that influence HIV/AIDS self-testing uptake and analyse the interaction between them, by taking into account unaddressed location variables, thus determining districts that require closer attention. Notably, this is the foundation for constructing a spatial dynamics model, as well as the basis for developing location and evidence-based intervention strategies. Although evidence from the literature, [24,25] provides the merits and acceptance of HIV/AIDS self-testing initiatives, there is a lack of evidence on modelling factors associated with HIV/AIDS self-testing uptake in South Africa. Understanding how geographical differences, as well as people’s attitudes and perception, influences HIV/AIDS self-testing uptake, may yield imperative location intelligence interventions, and tailor-made programmes to enhance uptake across the country. This study, therefore, seeks to extend the work of Jooste et al. [11] by exploring relationships and scale differences in people’s attitudes and perceptions of HIV/AIDS self-testing uptake using Generalised Linear Poisson Regression (GLPR) and Geographically Weighted Poisson Regression (GWPR) models. Revelations from this study will establish the existence or non-existence of spatial relationships between HIV/AIDS self-testing uptake and its related factors at subnational level.

## 2. Materials and Methods

### 2.1. Data

The study utilised the 2017 SABSSM V data [26] which was collected using a multistage stratified, cross-sectional national survey covering all age groups. Household samples were randomly selected from 1457 small area layers, using systematic probability sampling. The selected small area layers were extracted from 84,907 small area layers computed by Statistics South Africa in 2011 [27]. Small area layers were stratified by province using locality type, i.e., urban informal, urban formal, rural formal, and rural informal. Furthermore, a systematic random sample of 15 households was selected from each sampled small area layer. Remarkably, the household response rate from the survey was recorded at 82% and all household members participated in the survey [28]. The outcome variable for this study was the question: If an HIV self-test kit was available to you, would you be willing to use it to test yourself? It should be noted that the question was a categorical outcome variable coded as follows: 1 = yes; 2 = no; 3 = do not know, and for this study, “yes” was selected for the analysis. A set of explanatory variables (Table 1) was selected based on previous studies [4,16,17,24,29,30,31,32,33,34,35] to assess factors associated with HIV/AIDS self-testing uptake or willingness. Notably, explanatory variables used in this study were arrived at after multicollinearity testing, leaving variables with low correlation. The first question used as an explanatory variable for this study is (i) in general, would you say that your health is excellent, good, fair, or poor? The response to the question was a categorical outcome with responses: 1 = excellent, 2 = good, 3 = fair, 4 = poor, and then “excellent” was selected for the analysis. The second explanatory variable was based on the question: when was the last time you went to see a health professional? The responses to this question were coded as follows: 1 = within the past six months; 2 = more than six months but not more than a year ago, 3 = more than one year ago, and 4 = never. For the study codes 2 and 3 responses were combined for further analyses. The third question used as an explanatory variable was: what is the highest education level that you obtained? The responses included 17 different answers, with the study combining at least Grade 7 and up to Grade 12 for the analysis.

### 2.2. Spatial Autocorrelation—Global Moran’s I

The global spatial autocorrelation (Global Moran’s I) was computed to assess whether the district level spatial distribution of HIV/AIDS self-testing uptake and the covariates was dispersed, clustered, or random. Moran’s I [36], was utilised in this study to assess the presence or absence of spatial dependence and clustering of residuals (the index ranges between −1 and +1). When Moran’s I is positive, the distribution has a propensity towards clustering of similar values (+1), and 0 is usually indicative of no spatial autocorrelation. However, for a negative Moran’s I (−1), the distribution tends towards a perfect dispersion, with clustering of dissimilar values [36]. A detailed explanation of spatial autocorrelation using Global Moran’s’ I is published in multiple studies [10,37,38,39,40,41].

### 2.3. Generalised Linear Poisson Regression Modelling of Factors Associated with HIV/AIDS Self-Testing Uptake

When predicting discrete, non-negative, and non-continuous variables (count of responses from the SABSSM V, 2017), it is more appropriate to use Generalised Linear Models (GLM) to determine the relationship between outcome and explanatory variables [42]. However, GLM are spatially rigid and assume fixed effects for various locations in space. The models work on the assumption that a single equation can explain the same effect for all spatial units [42]. Given that variables used in this study are count data with discrete and non-negative values, the GLM was computed by implementing the Poisson distribution error [43]. GLPR explains the global relationship (Equation (1)) between HIV/AIDS self-testing uptake and the covariates (excellent health, more than 6 months after the last health professional visit and at least having attained Grade 7–12).

(1) $$In\left[E\left(y_{i}\right)\right]=\beta_{0}+\beta_{i}In\left(P_{i}\right)+\beta_{2}x_{2i}+\ldots +\beta_{k}x_{ki}+\varepsilon_{i}$$

where, $\left[E\left(y_{i}\right)\right]$ is the natural log of the expected count of HIV/AIDS self-testing uptake per district in the study area, $P_{i}$ is the offset variable, $x_{ki}$ is the *k*-th explanatory variable, $\beta_{i}$ is the *i*-th model parameter, index *i* refers to the district, and $\varepsilon_{i}$ is the *i*-th random error term.

### 2.4. Geographically Weighted Poisson Regression Modelling of Factors Associated with HIV/AIDS Self-Testing Uptake

GLPR is not capable of capturing spatial dependence in data, and it ignores the spatial correlation in the estimation of relationships [42,44]. Equally important, it is more unlikely that one single coefficient per explanatory variable can reflect the true spatial relationship between the dependent variable and the explanatory variable since spatial data vary in space [44,45]. This study explored the effectiveness of Geographically Weighted Regression (GWR) [46] that detects spatial heterogeneity in the dataset, relaxing the assumption of spatial stationarity associated with global models. When analysing spatially varied count data, it is plausible to utilise Poisson distribution within the GWR framework [40,47]. GWPR allows for each parameter to vary across the districts capturing important subnational level variations in the association between HIV/AIDS self-testing uptake and explanatory variables [48]. GWPR (Equation (2)) integrates GLM with GWR and extends the concept of the GWR models in the context of GLR [49]. The model was used to establish how relationships in HIV/AIDS self-testing uptake and the covariates differed at district levels in South Africa. More importantly, the model makes a great contribution to the development of South Africa, sub-national level HIV/AIDS self-testing uptake policies by specifying districts in most need of intervention rather than generalising effects of HIV/AIDS self-testing uptake for the entire country.

ln(*E*(*Yi*)) = *β*_0_(*s*) + *β*_1_(*s*)*x*_1_*_i_* + *β*_2_(*s*)*x*_2_*_i_* + … + *β_k_*(*s*)*x_ki_* (2)

where *Yi* is the observed count data at district locations *i*. *E*_(*Yi*)_ is the HIV/AIDS self-testing uptake at district locations *i*. *β*_0_(*s*), *β*_1_(*s*), *β*_2_(*s*), …, *β_k_*(*s*) are the spatially varying coefficients, which may differ across different districts. *x*_1*i*_, *x*_2*i*_, …, *x_ki_* are the predictor variables at district locations *i*. ln() is the natural logarithm. In this study, spatially varying coefficients are estimated using bi-square, kernel function that assigns weights to nearby observations. Thus, observations further from a particular district location have less influence on the estimation of the coefficients.

### 2.5. Model Diagnostic Indicators

The diagnosis of the GLRP model was established by analysing the pseudo R^2^ (Equation (3)), the Akaike information criterion (AICc) (Equation (4)) and deviance residuals.

(3) $$Pseudo\;R^{2}=1-\frac{D\left(y,y_{pred}\right)}{D\left(y,y_{mean}\right)}$$

where $D\left(y,y_{pred}\right)$ = deviance of the fitted nonlinear model, $D\left(y,y_{mean}\right)$ = deviance of the Intercept-only model.

The *AIC* (Equation (4)) that estimates the distance between a model and an ideal but unobservable model [50] was also utilised as the model diagnosis. A lower value for AICc is desired since it implies the most parsimonious model and the amount of variance that the model could not explain.

(4) $$AIC=-2\log\left(L\left(\hat{\theta }\right)\right)+2k$$

where *L*($\hat{\theta }$) is the maximum likelihood of estimated parameters ($\hat{\theta }$) given the data and model. ($\hat{\theta }$) quantifies the effects of explanatory variables on a model and include the intercept, the regression coefficients and the residual variance.

Overall model performance between GLPR and GWPR was established using local percent deviance explained. Percent deviance explained is comparable to the local determination coefficient (R^2^) and allows visualisation of spatial difference of the explanatory power of the model. Higher percent deviance values are more desirable. Furthermore, all analysis and maps for this study were computed and generated in ArcGIS Pro 3.0.2 (ESRI, Redlands, CA, USA).

## 3. Results

### 3.1. District Level Spatial Autocorrelation Assessment

**Figure 1:**
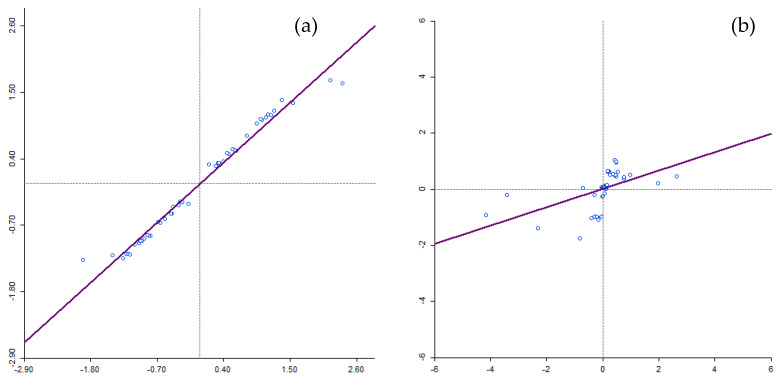
Moran’s I scatter plot for GLRP and GWPR models clustering between HIV/AIDS self-testing uptake and the covariates.

Moran’s I scatterplots (Figure 1) computed in this study sought to determine the clustering of residuals, modelling HIV/AIDS self-testing uptake, and the covariates using GLPR and GWPR. On the scatter plot, the upper-right quadrant (displays of positive Gi* statistics) and the lower-left quadrant (displays of negative Gi* statistics) correspond with positive spatial autocorrelation and on the contrary, the lower-right and upper-left quadrants correspond with negative spatial autocorrelation. Analysis of deviance residuals derived from GLRP, under the null hypothesis of no spatial autocorrelation, showed the presence of spatial autocorrelation. The output (Figure 1a) exhibited a degree of clustering (Moran’s’ I = 0.563; Z-Score = 3.149; *p*-value = 0.001) in the relationships between HIV/AIDS self-testing uptake with covariates used in the study (excellent health, more than 6 months after the last visit to a health professional and at least having attained Grade 7–12) from GLPR. Furthermore, as indicated from the output of the analysis of deviance residuals derived from GWPR using Moran’s I statistics (Moran’s’ I = −0.105; Z-Score = 0.980; *p*-value = 0.3268), the output illustrated a lack of spatial autocorrelation indicating spatial randomness (Figure 1b).

### 3.2. Generalised Linear Poisson Regression—Global Model

General statistics from the 2017 SABBSSM V survey revealed a high prevalence of HIV/AIDS self-testing uptake (N ranging between 1078 and 1692, total district counts) in Sedibeng, Gert Sibande, Uthukela, eThekwini, iLembe, and King Cetshwayo districts. On the contrary, Central Karoo and Namakwa districts recorded the lowest count of HIV/AIDS self-testing uptake ranging between 37 and 80. Model performance in terms of pseudo R^2^ and AICc recorded from GLRP resulted in percent deviance explained = 0.88 and AICc = 2552. Comparatively, results from the GWRP model showed better performance with a significantly smaller AICc = 390 and percent deviance explained R^2^ = 0.91 (Table 2).

**Figure 2:**
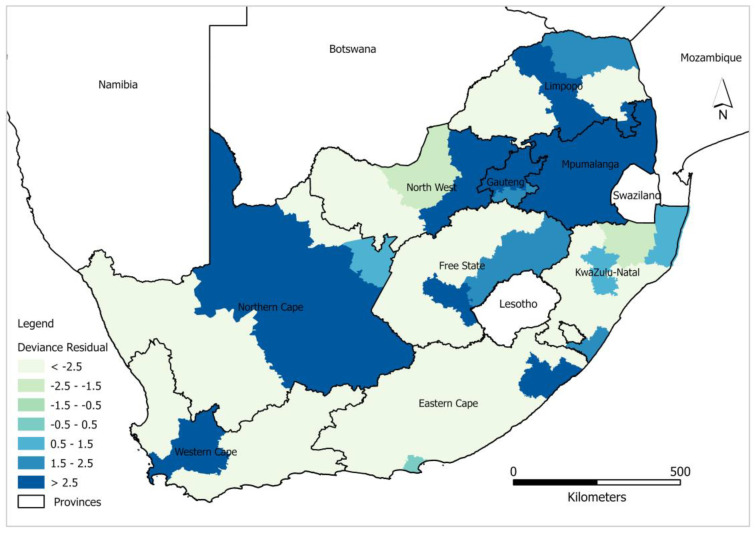
Spatial distribution of GLRP model deviance residuals.

Statistically significant district level clustering of deviance residual confirms spatial dependency of HIV/AIDS self-testing uptake prevalence using a global model. High negative clustering (deviance residuals < −2.5) was predominantly observed in the districts around Northern Cape, Western Cape, and Eastern Cape including Namakwa, West Coast, Garden Route, and Sarah Baartman districts. Contrary, high positive clustering (deviance residuals > 2.5) was observed in Capricorn Bojanala, City of Tshwane, City of Johannesburg, and Nkangala districts, etc. Theoretically, if the output of the spatial autocorrelation analysis exhibits a lack of spatial dependence, then the GLRP model is sufficient to explain the relationship between HIV/AIDS self-testing and covariates. However, from the output derived from the models, the spatial distribution of deviance residuals (Figure 2) showed evidence of spatial dependence. In light of this, our study further tested a more robust local model i.e., GWPR to account for the unexplained spatial lag. GWPR addresses spatial non-stationarity in the model by removing the limitations of a global model, allowing for local variance to be calculated in different districts. More specifically, GWPR allows regression coefficients to be calculated for different districts in contrast to GLRP which assumes a global fit for multiple geographic units at national level.

### 3.3. District Level Geographically Weighted Poisson Regression—Local Model

**Figure 3:**
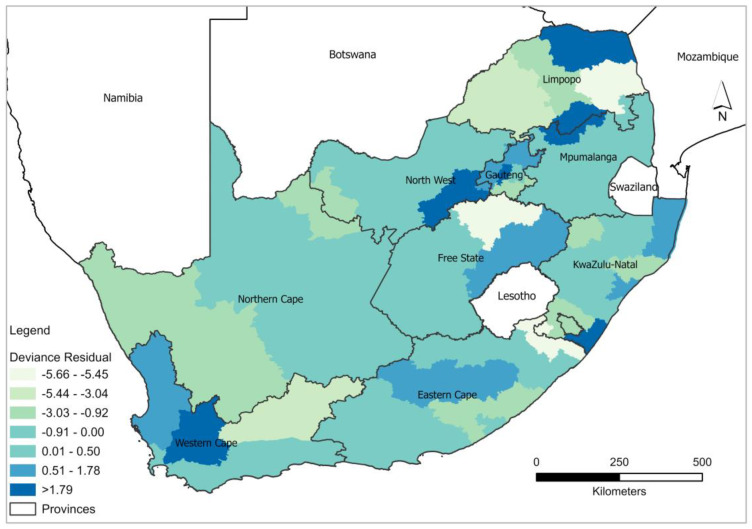
Spatial distribution of GWPR model deviance residuals.

GWPR was computed to analyse the influence of covariates on modelling HIV/AIDS self-testing uptake and the result showed a slight improvement in local model performance compared to the global model (Table 2). Furthermore, the percent deviance explained by the GWRP model indicates high rates of association between covariates (excellent health, more than 6 months after last visit to the health professional, and at least having attained Grade 7–12) and HIV/AIDS self-testing uptake. The deviance residual analysis of GWPR confirms the superior performance of the local model in comparison to the global model. The deviance residual from the estimated parameters of GWPR shows spatial independence (Figure 3). High negative deviance residuals ranging between −5.66 and −5.45) were observed in Mopani and Fezile Dabi districts around Limpopo and Free State. On the contrary high positive deviance residuals (1.79–3.67) were observed in Vhembe, Sekhukune (Limpopo), City of Johannesburg (Gauteng), Dr Kenneth Kaunda (North West), Ugu (Eastern Cape), and Cape Winelands (Western Cape). Overall, the results displayed in Figure 3 validate and indicate that GWPR explains better the variability in the data and provides favourable results for explaining the relationship between HIV/AIDS self-testing uptake and the covariates in different districts.

**Figure 4:**
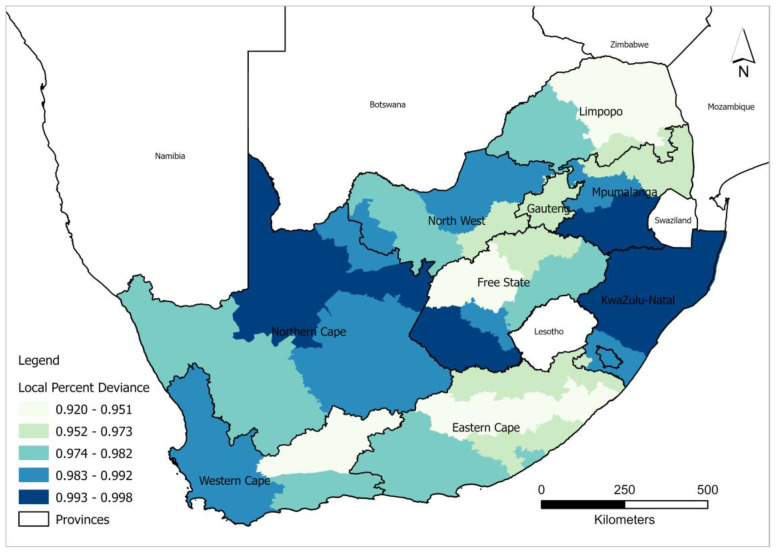
Spatial distribution of local percent deviance from the GWRP model.

The local percent deviance explained shows spatial variation in the explanatory power of the GWPR model across different districts in South Africa in terms of predicting the relationship between HIV/AIDS self-testing uptake and the covariates (Figure 4). A significantly lower AICc is indicative of the existence of heterogeneity among different explanatory features and this heterogeneity, however, cannot be captured by the global model. The local percent deviance ranged between 90.2 and 99.7 and all the districts show the best fit (greater than 90 percent deviance explained) which best explains the relationship between HIV/AIDS self-testing uptake. The low predictive power was recorded and well distributed in the districts around Limpopo such as Vhembe, Capricorn, and Mopani; Lejweleputswa in Free State; Central Karoo in the Western Cape; and Chris Hani alongside O.R. Tambo, in the Eastern Cape. In contrast, very high percent deviance values were recorded in ZF Mgcawu, in the Northern Cape, and most districts in KwaZulu Natal including Zululand, Amajuba, King Cetshwayo, Lembe, eThekwini, and Umkhanyakude.

**Figure 5:**
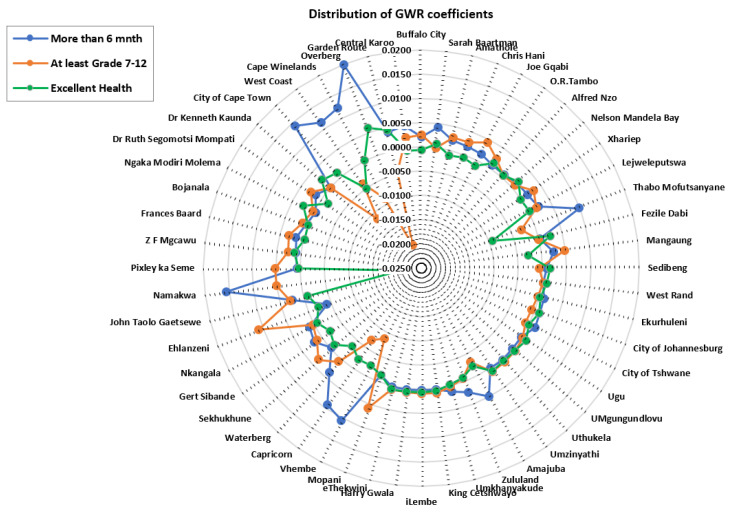
Spatial distribution of coefficients derived from the GWRP model.

GWPR coefficients (Figure 5) present negative and positive strengths of covariates used in the study, thus providing insight into district level targeted interventions. The difference in the associations between HIV/AIDS self-testing uptake and the covariates: at least having attained Grade 7–12, excellent health, and more than 6 months after the last visit to the health professional was observed across different districts (−0.0042–0.0109). Coefficients of the covariate: at least having attained Grade 7–12 showed significantly negative strength (−0.0199–−0.0069) to HIV/AIDS self-testing in the Western Cape (Overberg, Cape Winelands, City of Cape Town, and Eden) and Limpopo (Vhembe and Capricorn). In addition, “having spent more than 6 months after the last visit to the health professional” resulted in negative coefficient values (−0.0042–−0.0041) in Mopani, Pixley ka Seme, Bojanala, and Zululand districts, etc. Excellent health variables exhibited negative strength (−0.0207–−0.0017) in the Western Cape district (Namakwa) which was comparable to the variable “having at least attained Grade 7–12”. Positive strength of covariates (0.0008–0.0200) is more prevalent for “more than 6 months after the last visit to the health professional” which is indicative of greater association to HIV/AIDS self-testing uptake, and this was followed by “having at least Grade 7–12”. Excellent health exhibited a more negative relationship with the HIV/AIDS self-testing, in comparison to the coefficient of the other two variables (more than 6 months after the last visit to the health professional and having at least attained Grade 7–12).

## 4. Discussion

This study explored the effectiveness of GLPR and GWPR models in examining the spatial dependence and non-stationary relationships of HIV/AIDS self-testing uptake and the covariates. Output from the study shows that 12% of the relationship between HIV/AIDS self-testing uptake and the covariates used in the study cannot be captured by the global model, compared to 9% by the local model. The results show the inefficiency of the global model due to the non-consideration of the scale of geographical processes associated with HIV/AIDS self-testing uptake. Local regression models capture local spatial disparities in the relationships between outcome and explanatory variables [51]. This is confirmed by the output of our study, modelling spatial differences in HIV/AIDS self-testing uptake with GWPR resulting in deviance variation percentages between 92 and 99, which is indicative of divergence in HIV/AIDS self-testing uptake variation across the country (Figure 4). GLPR showed a high propensity of both overestimation and underestimation in the northern part of the country and more overestimation in the western and eastern parts of the country. On the contrary, GWPR, exhibited a random pattern, with values close to −0.9 to 0.5 across the country. Moreover, the high deviance residual from the global model is indicative of the predicted value of the response variable being significantly different from the observed value, attributed to outliers around Northern Cape, Western Cape, Gauteng, and Limpopo (Figure 2). The variance in the response variable was not constant across the range of covariates used in the study (at least having attained Grade 7–12, excellent health, and more than 6 months after the last visit to the health professional).

HIV/AIDS self-testing has been recognised in the literature as having significant advantage of increased privacy and confidentiality, as well as convenience compared to conventional HIV/AIDS testing administered by health professionals [4,17,24,25,28,30,52,53]. Previous studies considered various methodological approaches in documenting, mapping and quantifying HIV/AIDS self-testing uptake [2,4,30,52]. However, such studies have been unable to factor in the presence of spatial autocorrelation and the unobserved heterogeneity that might appear in the area of interest. More specifically, the relationship between the outcome and explanatory variables is assumed to be stationary in space [46]. Results from this study showed GLPR = 88% deviance versus 91% for GWPR, which was a slight increase in the performance of the local model compared to the global model (Table 1). Better performance of GWPR can be attributed to geographical variation in South Africa [54], which can influence access to healthcare facilities as well as access to education. More importantly, differences in access to infrastructure (road, water, electricity, and health facilities) around urban and rural districts widen the disparities, observed from the outcome of the study [8,55]. Furthermore, due to urbanisation and conflict across the globe, migration contributes to diverse cultures which to some extent influence people’s attitudes and knowledge in society towards HIV/AIDS self-testing uptake [56].

Local percent deviance differed across districts illustrating the difference in the combined statistical impact of the local model variables and this could be attributed to unique demographic characteristics. Similar observations were reported by Jooste et al. [11] who mapped the uptake of HIV testing at the district level in South Africa. They noted that differences in demographic distribution across the districts in South Africa influenced the observed proportions of people accepting HIV/AIDS self-testing. In a separate study, Sambisa et al. [52] reported a higher prevalence of self-reported HIV testing among women in comparison to men in Zimbabwe, and this was due to a different population count of men and women in the various communities. Furthermore, the disparity in acceptance of HIV testing between women and men was attributed to initiatives and programmes targeted at the former, which are in line with the prevention of mother-to-child HIV transmission. Further explanation of the differences in uptake levels reported in this study could be due to HIV/AIDS positive status stigmatisation. Similar conclusions were arrived at by Sonko et al., Berendes and Rimal, and Sambisa et al. [29,35,52] who noted that individuals who knew anyone with HIV/AIDS experiencing stigmatisation, would be reluctant to test and subsequently know their status. On the contrary, some people would volunteer to have HIV/AIDS self-testing in order to obtain immediate medical assistance if found positive. Output from this study should set as a benchmark for responsible authorities and various stakeholders to support widespread initiatives of HIV/AIDS self-testing uptake in geographical locations that exhibit low uptake. The campaign should encourage the general population to test, thus enabling individuals to know their status hence reducing undiagnosed HIV/AIDS infections among the general population. Equally important, the creation of more targeted policies to increase HIV/AIDS self-testing uptake can be afforded by location intelligence derived from Geographic Information System based techniques [37].

With notable variance in the strength of GWPR coefficients, the study identified districts with both significant positive and negative relationships between each explanatory variable and HIV/AIDS self-testing uptake. Figure 5 highlights comparisons of covariates (at least having attained Grade 7–12, more than 6 months after having visited a health professional and excellent health) in explaining the model. A negative relationship of “at least having attained Grade 7–12” with HIV/AIDS self-testing uptake was prevalent in districts around Limpopo (Vhembe and Capricorn) and Western Cape (Overberg and City of Cape Town). This reveals the relatively weak prediction power of (having attained at least Grade 7–12) education level in explaining HIV/AIDS self-testing uptake. It is imperative to focus on initiatives targeting educational institutions specifically for Grade 7–12 students in those districts. The authors of [2] associated socioeconomic differences with the uptake of HIV/AIDS testing in South Africa and reported an increased likelihood of testing from respondents with secondary education. Such remarks show low uptake in populations with limited education attributed to possible ignorance in interpreting the self-testing results and potentially making mistakes throughout the whole process of self-testing. Overall, HIV/AIDS self-testing services must be accessible to age groups engaged in sexual activities. Attaining at least Grade 7–12 improves one’s HIV/AIDS knowledge, since Grade 7–12 curriculum covers aspects related to sex education and there would be discussions on the importance of HIV/AIDS status awareness [35]. A significant positive association was established in Namakwa, Pixley ka Seme, Mopani, Sekhukune, and Ehlanzeni districts. This result shows that various individuals feel confident enough to perform self-testing with the confidence of being error-free and having enough knowledge to carefully read and interpret the results.

The positive association between more than 6 months since the last healthcare profession visit and HIV/AIDS self-testing highlighted in districts around Northern Cape (Namakwa, ZF Mgcawu and Pixley ka Seme) Eastern Cape (Sarah Baartman, Nelson Mandela Bay, and Amathole, etc.), and Western Cape (Garden Route, City of Cape Town, and West Coast) could be highlighting good health amongst respondents, hence less frequent visits to medical professionals. Further explanation of the positive association between HIV/AIDS self-testing and fewer healthcare professional visits could be associated with lifestyle and access to healthy food items thus boosting their immune system. This is more apparent in urban districts (City of Cape Town and Nelson Mandela Bay), where access to nutritious retail food items is better compared to the rural districts [54]. On the contrary, Jin et al. [57] postulated that poor attitudes by some healthcare professionals contribute to hesitancy in frequent visits to healthcare professionals. In addition, the fear of HIV/AIDS-positive test results exerts influence on people to only seek healthcare when they are sick. Results from the GWPR model showed a negative relationship between HIV/AIDS self-testing uptake and respondents with more than 6 months after their last health professional visit in districts around KwaZulu Natal (Ugu, eThekwini, Zululand, and Umkhanyakude, etc.) and Northern Cape (Bojanala, Dr Ruth Segomotsi, and Dr Kenneth Kaunda). Non-metropolitan districts have poor transport facilities, for instance, in Ugu district, the average travel distance to the closest hospital is 4 km and in Umkhanyakude, it is 6.3 km. ZF Mgcawu and Pixley ka Seme are constrained by poor road networks; hence, distance to the closest clinic could also play a role in determining the frequency of visits, because people have to travel very long distances [58]. Geoterra Image data (Geoterra image 2018) show an average travel distance of 10.6 km to the closest hospital in Pixley ka Seme (rural district), while in Cape Town (urban district), the distance is 0.75 km. Another explanation for the negative association of HIV/AIDS self-testing and having more than 6 months after the last healthcare professional visit could be attributed to the higher costs of medical consultation fees, limiting people to fewer healthcare professional visits.

Perceptions of excellent health status can convince people to have a negative attitude towards acceptance of HIV/AIDS self-testing. Excellent health status is regarded as an essential indicator of satisfaction with life in relation to any illnesses one can succumb to. This subjective perception of an individual’s sense of excellent well-being, with the absence of feelings of discomfort or pain and psychological consequences of having a health problem, contributes to hesitance in HIV/AIDS self-testing initiatives [29]. Negative association in perception of excellent health with HIV/AIDS self-testing uptake is more prevalent around various districts across the country including West Coast, Namakwa, Vhembe, Capricorn, Waterberg, Mangaung, Gert Sibande, and Thabo Mofutsanyane. The lack of a strong relationship between the perception of excellent health and HIV/AIDS self-testing uptake could be attributed to fear of denunciation and a loss of emotional control because of the mental burden of knowing one’s HIV/AIDS positive status [11,20]. Equally important, the fear of having a positive result without any immediate personal support can be devastating to people, thus the increased hesitancy in self-testing uptake [24,25].

Some of the limitations of this current study include the following. The cross-sectional design of the survey limits the causal inferences between HIV/AIDS self-testing uptake with the analysed covariates. Thus, there may be a high degree of municipal district heterogeneity. The findings of this study should only be explained at the county-scale level and local inferences can be misleading/inaccurate due to ecological fallacy.

## 5. Conclusions

This study demonstrated the efficacy of GWPR in modelling HIV/AIDS self-testing uptake and its associated covariates. It has been noted that HIV/AIDS self-testing uptake provides a chance for individual testing to be performed inconspicuously, at one’s convenience. GWPR analysis enables the identification of the specific predictors at each local and specific district municipality. Modelling the uptake of HIV/AIDS self-testing enables targeted interventions on reluctant individuals, by developing evidence-based and location intelligence interventions for areas in most need. The contributing factors analysed in this study, i.e., excellent health, more than 6 months since last health professional visit, and at least having attained Grade 7–12, showed mixed effects in explaining HIV/AIDS self-testing uptake. Several barriers continue to limit the full adoption of HIV/AIDS self-testing with issues raised around the absence of counselling after testing, and challenges in the accurate use of HIV/AIDS kits compromising the reliability of the results. South Africa has a diverse population; thus, the provision of HIV/AIDS self-testing programmes should consider the context and culture of the target populations and that some population groups would prefer the use of self-testing kits to avoid the need for counselling which might be futile to them. HIV/AIDS self-testing uptake is correlated with interpersonal, sociocultural, and economic factors in mostly nonlinear ways because it is context related. Therefore, future research should be aimed at using spatial statistics and other various statistical techniques that include the spatiotemporal dimension and exploring other factors that influence HIV/AIDS self-testing uptake across South Africa. This will in turn enable meaningful and geotargeted interventions, thus making better use of limited resources.

## Figures and Tables

**Table 1:** Outcome and explanatory variables used in the study.

	Variable	Description of Covariates
Outcome Variable	Self-testing uptake	If an HIV self-test kit was available to you, would you be willing to use it to test yourself?
Explanatory Variables	Excellent health	In general, would you say that your health is excellent?
	More than 6 months	When was the last time you went to see a health professional?
	At least Grade 7 up to Grade 12	What is the highest educational level that you obtained?

**Table 2:** Comparison of model performance.

Model Type	AICc	Percentage Deviance Explained
Global Model—GLRP	2552	0.88
Local Model—GWRP	390	0.91

## Data Availability

Data used in this analysis are available from HSRC’s public data repository (data set). SABSSM 2017 Combined. Version 1.0. Pretoria South Africa: Human Sciences Research Council (producer) 2017, Human Sciences Research Council (distributor) 2020. https://doi.org/10.14749/1585345902 (accessed on 10 September 2022). Archive number: SABSSM 2017 Combined, doi:10.14749/1585345902.
